# Potato Preload Mitigated Postprandial Glycemic Excursion in Healthy Subjects: An Acute Randomized Trial

**DOI:** 10.3390/nu12092759

**Published:** 2020-09-10

**Authors:** Wenqi Zhao, Ying Zhou, Yuqing Yuan, Zhihong Fan, Yixue Wu, Anshu Liu, Xuejiao Lu

**Affiliations:** 1 College of Food Science and Nutritional Engineering, China Agricultural University, Beijing 100083, China; zhaowenqi@cau.edu.cn (W.Z.); hpyzyzy@163.com (Y.Z.); yuanyuqing0902@126.com (Y.Y.); xiaoc0105@126.com (Y.W.); liuanshu@cau.edu.cn (A.L.); feirlu@163.com (X.L.); 2 Key Laboratory of Precision Nutrition and Food Quality, Department of Nutrition and Health, China Agricultural University, Beijing 100083, China

**Keywords:** glycemic response, preload, potato, partially hydrolyzed guar gum, satiety

## Abstract

This study investigated the preload effect of the medium and high glycemic index (GI) potato, as well as the combination of partially hydrolyzed guar gum (HG) and potato, when ingested prior to a rice meal, on the iso-carbohydrate basis. In a randomized crossover trial, 17 healthy female subjects consumed (1) rice; (2) co-ingestion of highly cooked potato (HP), and rice (HP + R); (3) co-ingestion of minimally cooked potato (MP) and rice (MP + R); (4) preload HP prior to rice meal (PHP + R); (5) preload MP prior to rice meal (PMP + R); (6) co-ingestion of partially hydrolyzed guar gum (HG), HP and rice (HG + HP + R); (7) preload HG prior to co-ingestion of HP and rice (PHG + HP + R); (8) co-preload of HG and HP prior to rice (PHG + PHP + R); and (9) preload of HP prior to co-ingestion of HG and rice (PHP + HG + R). Postprandial glycemic response (GR) tests and subjective satiety tests were conducted for each test food. Cooked potato as a preload to a rice meal could significantly cut the acute postprandial glycemic excursion by around 1.0 mmol/L, irrespective of the GI of the preload. Co-preload of partial hydrolyzed guar gum and highly cooked potato (PHG + PHP + R) resulted in improved acute GR in terms of peak glucose value and glycemic excursion compared with either HG preload or HP preload. All the meals with preload showed comparable or improved self-reported satiety. Within an equicarbohydrate exchange framework, both high-GI and medium-GI potato preload decreased the postprandial glycemic excursion in young healthy female subjects. The combination of HG and HP as double preload resulted in better GR than both single HG or HP preload did.

## 1. Introduction

The prevention and control of diabetes and its complications have been attached with great importance, as the global prevalence of global diabetes reached 463 million in 2019 and is estimated to rise to 700 million by 2045 [1]. In addition to medication, mounting evidence showed that dietary intervention could play a vital role on glycemic management and diabetes prevention [2].

As one of practicable postprandial glycemic attenuating strategies, preliminary studies indicated that preload interventions resulted in curbed glycemic excursion as well as favorable insulin and gut hormone responses [3,4]. In addition to the protein preload, previous studies suggested that the preload of carbohydrate-rich food, such as rice [5], fruits [5,6], and sugar solution [6], could yield more stable postprandial glycemic responses (GRs) compared with their non-preload counterparts.

It is generally acknowledged that low glycemic index (GI) meals are associated with enhanced glycemic control and insulin sensitivity, while high GI diets elicited adverse metabolic effects in the diabetic and prediabetic groups [7,8]. However, it has not yet been confirmed whether the hypoglycemic effect of carbohydrate preload is a universal rule that can be applied to more carbohydrate food including potatoes. In addition, whether the GI of carbohydrate preload can affect its glycemic effect is still unknown.

Potato (*Solanum tuberosum* L.) is consumed worldwide either as starchy vegetables, snacks, or staple foods. Roots and tubers rank as the third largest carbohydrate food source in the world while the potato accounts for nearly half of all root consumption [9]. A meta-analysis of epidemiological studies suggested a positive association between potatoes consumption and risk of type 2 diabetes [10], but many studies showed that the glycemic characteristics of potato foods varied substantially among cultivars [11]. Non-fried potato is regarded as a food of high satiety index [12], and minimal processed potato can be a good dietary source of vitamin C, several B vitamins, and polyphenols [9], as well as resistant starch (RS) [13]. A 14-year follow up research reported that potato consumption was associated with lower all-cause mortality when substituting grains in Chinese diet [14]. A recent randomized crossover intervention study indicated that four-week consumption of non-fried potato as a side dish did not affect glycemic markers in healthy female subjects [15]. These findings justified more exploration on the metabolic consequences of potato foods.

Microwaving is an efficient way of cooking potato with less nutrient loss compared with steaming and boiling [16,17]. The intensity of microwave heating might have an impact on subsequent GRs of potato food, as the treatment had profound effects on starch granule and tissue characteristics [18,19]. By adjusting the heating parameters, it might be feasible to manipulate the glycemic characteristics of potato samples without affecting their macronutrient composition.

High viscosity soluble fiber, such as guar gum, is believed to be able to improve postprandial glycemic response of high GI meals [20]. However, high amount of high-viscosity dietary fiber might not be easily tolerated as a regular preload in daily life. The partially hydrolyzed guar gum (PHGG, further abbreviated as HG here for convenience) is a low-viscosity soluble dietary fiber derived from galactomannan, with an average molecular weight of 20 kDa, which has little impact on physical properties of food. Limited studies showed that the addition of HG to diet might have favorable effect on diarrhea [21], blood lipids [22], intestinal microbiota [23]. and postprandial GRs [24]. There is the possibility that the combination of a soluble dietary fiber, such as HG and carbohydrate food, has a synergistic preload effect in terms of postprandial glycemic and satiety characteristics of high GI meals.

This study attempted to investigate the preload effect of the medium and high GI potato in young healthy females, as well as the combination of HG and potato, when ingested prior to a rice meal, on the iso-carbohydrate basis. We assumed that (1) the potato preload could mitigate postprandial GRs compared with its co-ingestion counterparts; (2) the GI of the preload potato might make a difference in its GR effects; (3) the preload treatment would not negatively affect the subjective satiety; and (4) preload of hydrolyzed guar gum and high GI potato might have a synergy effect and achieve a better GRs than the single-preload counterparts.

## 2. Materials and Methods

### 2.1. Subjects Recruitment

Healthy female university students who met the following criteria were recruited via advertisement and moments: (1) no metabolic diseases, including but not limited to impaired glucose tolerance, diabetes, hypertension, hyperuricemia and dyslipidemia; (2) body mass index (BMI) within the range of 18.5~24.0; (3) not a regular alcohol drinker, smoker; (4) no dependency on medication or drugs; (5) not in pregnancy or lactation; (6) free from acute or chronic allergies; (7) no diagnosed digestive disorders or self-reported frequent gastrointestinal upset; (8) regular consumption of three meals a day; (9) not on weight loss in the past three months; and (10) no eating disorders, such as anorexia nervosa, bulimia, and binge eating.

After a duplicated oral glucose tolerance test (OGTT), 17 eligible volunteers signed the informed consent forms and complete all the test procedures. Carried out at China Agricultural University, the study was registered on the Chinese Clinical Trial Registry (registration number ChiCTR2000033216) and approved by the Ethics Committee of China Agricultural University (ethics number CAUHR-2019007).

### 2.2. Study Design

Referring to the Englyst method [25], in vitro starch digestion experiments were carried out to determine the microwave heating duration of potatoes as the test food in subsequent human trials. The human study was performed as a randomized, crossover, acute feeding design and the sample size was verified by PASS 13 Power Analysis and Sample Size software (NCSS, Kaysville, UT, USA) calculation. With a coordinator blinded to the treatment performing the randomization, the subjects were assigned to 13 test meals separated by at least one week to minimize any carryover effects. The test sessions were not carried out in three days before the menstrual period and the 1~3 day of the period to avoid the possible impact of premenstrual syndrome.

In the day prior to the intervention, the subjects were asked to keep their usual physical and eating habits while rule out any alcoholic or caffeine-containing beverages and avoid staying up late and intense activity. After an overnight fast of around 12 h, participants arrived at the laboratory at 7:50 am and allowed to rest for 10 min before the first finger-prick. Then they were given their test meals and were instructed to consume the provided preload food within 5 min and the rice meal within 10 min. They were supplied with 150 mL plain water at 60 min after the rice meal. The participants were instructed to stay sedentary and not to discuss any food-related topics throughout the sessions.

Each subject was served with the following glucose references and test meals in three groups of study.

Study 1: (1) glucose solution; (2) highly cooked potato (HP); (3) minimally cooked potato (MP). The HP, MP and glucose were given in servings containing 25 g available carbohydrates (AC) to determine the GI of cooked samples used in group 2 tests.

Study 2: (4) glucose solution containing 50 g AC; (5) white rice containing 50 g AC; (6) preload HP 30 min prior to rice ingestion (PHP + R); (7) preload MP 30 min prior to rice ingestion (PMP + R); (8) co-ingestion HP with rice (HP + R); (9) co-ingestion MP with rice (MP + R). The preload effect of HP and MP was determined on the basis of isocarbohydrate load of 50 g AC, while the potato contributed 15 g AC and rice contributed the rest.

Study 3: (10) co-ingestion partially hydrolyzed guar gum, HP and rice (HG + HP + R); (11) preload HG, with HP and rice co-ingested 30 min later (PHG + HP + R); (12) preload HP, with HG and rice co-ingested 30 min later (PHP + HG + R); (13) preload HP and HG, with rice ingested 30 min later (PHG + PHP + R). The combination effect of HP and HG was determined on the basis of isocarbohydrate load of 50 g AC.

### 2.3. Meal Preparation

Peeled potatoes were cut into 1 cubic centimeter pieces, placed in Snap Boxes with 30.0 g plain water and cooked in microwave under conditions determined by in vitro carbohydrate digestion experiments. HG solution was made by 11.8 partially hydrolyzed guar gum powder completely dissolving in 100.0 g water in room temperature. The glucose (50 g AC) and rice (50 g AC) were prepared as dual reference foods. The weights of test meals were balanced by plain water. The potato and rice were cooked in the morning of the trial day and served at 50 °C to avoid possible retrogradation of starch.

HG (brand “Guarrun”) was produced by guarrun Co., Ltd., Beijing, China. Fresh potatoes (*Solanum tuberosum* L.) and the polished rice (*Oryza sativa* spp. *japonica*) were bought from the local supermarket, cultivated in Shandong and Heilongjiang, China, respectively. The nutritional composition of the test meals is shown in Table 1.

### 2.4. Measurements of Blood Glucose and Satiety

Blood glucose concentrations were determined by the glucose oxidase method. Finger pricking blood samples were obtained by glucometer (LifeScan Inc., Milpitas, CA, USA) before each meal and at 15, 30, 45, 60, 90, 120, 150, 180, 240 min after the start of the meal. Additional blood samples at 15 and 30 min following preload ingestion were also collected for the preload treatments.

Subjective appetite sensations were assessed by visual analogue scale (VAS) with length of 100 mm anchored by “not at all” on one side and “conceivable extremely” on the other side [26,27] at each blood-collecting time.

### 2.5. Data Processing and Statistical Analysis

Rapidly digestible starch (RDS), slowly digestible starch (SDS), resistant starch (RS), and starch digestion index (SDI) were calculated [28] to predict possible GRs. Indices of glycemic variability included the incremental peak (∆Peak) and low (∆Low) of blood glucose concentrations, the maximum amplitudes of glucose excursion (MAGE), baseline recovery 1 h after peak (−∆G/∆G) [29], continuous overall net glycemic action (CONGA_1_) defined as the standard deviation of all the differences between the current observation and the observation at 1 h previously [30], and the positive increments under the curve of postprandial GRs (iAUC) calculated by trapezoid summation [31]. The satiety index (SI) of each meal was calculated and defined as the iAUC_0−240_ generated by 50 g of available carbohydrate of the test meal, expressed as a percentage of that elicited by 50 g of rice [26].

Original data were entered into Microsoft Excel spreadsheets for preliminary collation. Treatment × time effects among test meals were assessed by two-factor repeated-measures ANOVA. One-way analysis of variance ANOVA was used for mean values statistical analysis and Duncan’s multiple range test was chosen to carry out the multiple comparisons test. The statistical analysis was performed using the SPSS version 21.0 (SPSS Inc. Chicago, IL, USA) and the figures were generated from Origin 8.5 (OriginLab Inc. Northampton, MA, USA).

## 3. Results

### 3.1. In Vitro Starch Digestion of Cooked Potato

**Figure 1:**
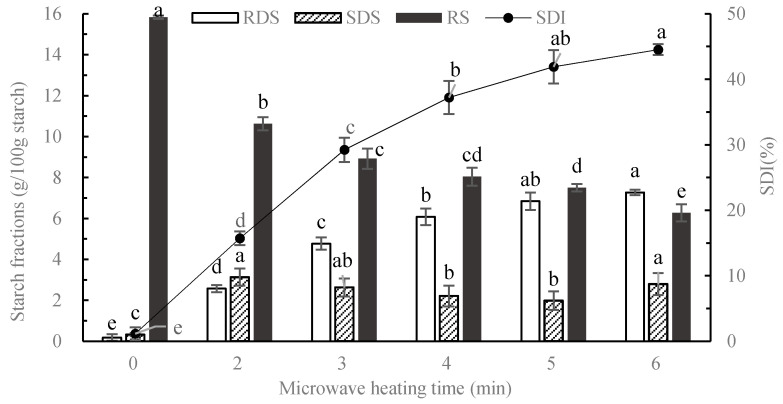
The starch digestion of potatoes heated by microwave for different durations. RDS, rapidly digestible starch; SDS, slowly digestible starch; RS, resistant starch; SDI, starch digestion index. Data were analyzed by one-way (treatment) ANOVA with Duncan’s multiple range test, significant differences (*p* < 0.05) were represented by different letters, vertical bars showed the standard errors of six replicates.

The starch fractions and SDI of potato samples microwave cooked for 0, 2, 3, 4, 5, 6 min are shown in Figure 1. After cooking for 7 min, the potato blocks were scorched on the edge and shrunken severely, so the maximum cooking time was 6 min. The shortest cooking time was set as 2 min according to sensory acceptability. With the extension of microwave cooking duration, the RDS fraction increased and the RS decreased. Taking the disparity of starch digestion and sensory acceptance into consideration, samples heated for 2 min and 6 min were chosen for subsequent human trials as highly cooked potato (HP) and minimally cooked potato (MP).

### 3.2. Glycemic Responses

Baseline anthropometric characteristics included: aged 20~24, mean (SD) BMI of 20.3 (1.6) kg/m^2^, fat mass of 28.0 (2.7)%, visceral fat index of 2.5 (1.4), basal metabolism rate of 1195.4 (87.0) kcal/day, mean (SE) blood glucose level of 5.1 (0.1), 7.2 (0.4), and 4.8 (0.1) at fasting, 1 h and 2 h in OGTT. All test data of 17 volunteers were put into analysis. The subjects did not report any adverse effect or unpleasant feeling during all test sessions.

#### 3.2.1. Glycemic Responses of Cooked Potatoes

**Figure 2:**
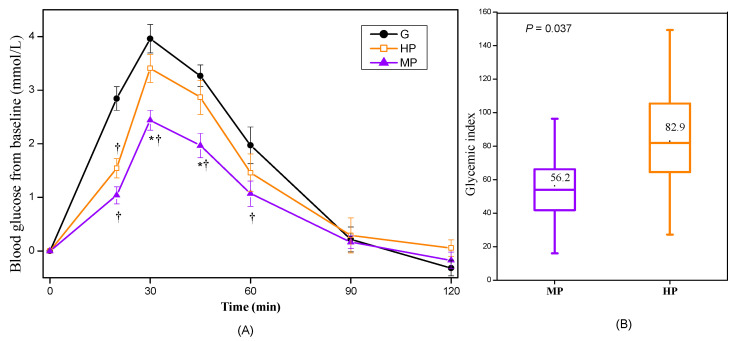
Glycemic responses of cooked potato samples (n = 17). G, glucose; HP, highly cooked potato; MP, minimally cooked potato. (**A**) Blood glucose changes from baseline for potato samples. (**B**) The GI values of potato samples. Values are shown as the mean value with their standard errors represented by vertical bars. * HP different from MP, ^†^ HP, or MP different from G (*p* < 0.05).

Figure 2 shows the GRs of HP and MP samples containing 25 g of available carbohydrates in study 1. The HP elicited significant higher glucose level at 30 and 45 min compared with the MP. The GI values of HP and MP, which were 82.9 and 56.2, had significantly difference (*p* = 0.037) and could be classified as high GI and medium GI food [32], respectively.

#### 3.2.2. Glycemic Response of Mixed Meals Containing Potatoes and Rice

**Figure 3:**
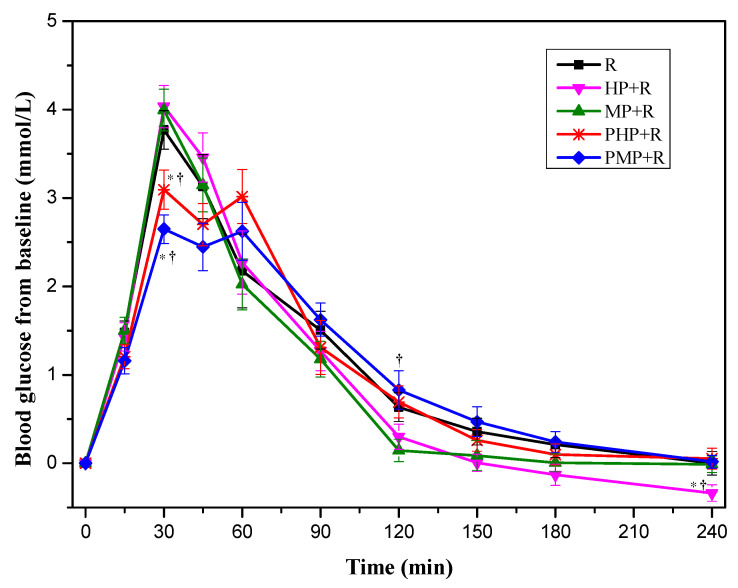
Blood glucose changes from baseline for mixed test foods (n = 17). R, rice; HP, highly cooked potatoes; MP, minimally cooked potatoes. HP + R, co-ingestion of HP and rice; MP + R, co-ingestion of MP and rice; PHP + R, preload HP with rice; PMP + R, preload MP with rice. Values are shown as the mean value with their standard errors represented by vertical bars. * Test meals different from rice, ^†^ preload different from co-ingestion test meals (*p* < 0.05).

In study 2, as shown in Figure 3, both PHP + R and PMP + R elicited significantly lower blood glucose increments compared with their corresponding co-ingestion treatments and R at 30 min. PMP + R resulted higher glycemic increments compared with MP + R at 120 min. The glycemic increments of HP + R is lower than that of PHP + R and R reference at 240 min. No significant difference was found between potato samples with different GI values at same ingestion condition in terms of glycemic increments.

Table 2 shows the indices of glycemic variability for test meals in 240 min. HP + R and MP + R showed comparable GRs compared with R reference except for that HP + R elicited smaller iAUC_120–240_ than that of R. The PMP + R resulted a significant reduction of peak value and MAGE compared with MP + R and R reference. The PHP + R showed lower value of MAGE and higher value of ∆Low than that of HP + R. The −∆G/∆G of PMP + R was smaller than that of PHP + R, which demonstrated a slower baseline recovery within 1 h after peak appearance. The PMP + R mitigated GRs in terms of iAUC_0–30_ and iAUC_0–60_ compared with MP + R and R, while no difference was found for iAUC_120–240_ and iAUC_0–240_.

#### 3.2.3. Glycemic Response of Mixed Meals Containing HG, Potatoes and Rice

**Figure 4:**
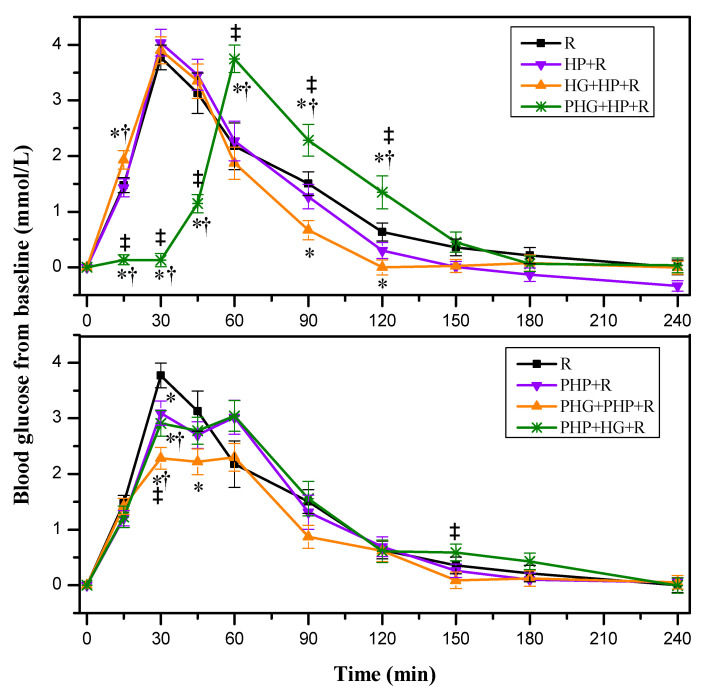
Blood glucose changes from baseline for mixed test foods (n = 17). R, rice; HP, highly cooked potato; PHP, preload HP; HG, partially hydrolyzed guar gum; PHG, preload HG; HP + R, co-ingestion of HP and rice; HG + HP + R, co-ingestion of HG, HP, and rice; PHG + HP + R, preload HG 30 min prior to co-ingestion of HP and rice; PHP + R, preload HP 30 min prior to rice; PHG + PHP + R, co-preload HG and HP 30 min prior to rice; PHP + HG + R, preload HP 30 min prior to co-ingestion of HG and rice. Values are shown as the mean value with their standard errors represented by vertical bars. * Test meals different from rice, ^†^ HG containing meal different from HP + R or PHP + R; ^‡^ HG preload meal different from HG post-ingested meal (*p* < 0.05).

As shown in Figure 4, taking the HP + R as a reference, adding co-ingested HG (i.e., HG + HP + R) elicited higher blood glucose increments compared with HP + R and R at 15 min and lower blood glucose increments compared with R at 90 and 120 min. However, adding preload HG (i.e., PHG + HP + R) resulted lower glycemic increments at 15, 30, 45 min and higher glycemic increments at 60, 90, 120 min compared with R, HP + R, and HG + HP + R, along with a delayed peak. On the basis of PHP + R, adding either preload or co-ingested HG (i.e., PHG + PHP + R or PHP + HG + R) showed lower glycemic increments at 30 min than that of R and PHP + R. Compared with PHP + HG + R, the glycemic increments of PHG + PHP + R was lower at both 30 min and 150 min.

As shown in Table 3, adding co-ingested HG to HP + R (i.e., HG + HP + R) showed no difference with R and HP + R in terms of indices of glycemic variability except −∆G/∆G, which was higher than that of R. However, adding preload HG to HP + R (i.e., PHG + HP + R) elicited lower MAGE than that of HP + R and higher CONGA_1_ than that of R, HP + R and HG + HP + R. Meanwhile, the PHG + HP + R resulted lower iAUC_0–30_, iAUC_0–60_ and higher iAUC_120–240_ than that of HP + R and HG + HP + R. On the basis of PHP + R, adding co-ingested HG (i.e., PHP + HG + R) showed no difference with HP + R, while adding preload HG (i.e., PHG + PHP + R) mitigated GRs in terms of ∆Peak, MAGE, and iAUC_0–240_ compared with PHP + R and PHP + HG + R.

### 3.3. Subjective Appetite

**Figure 5:**
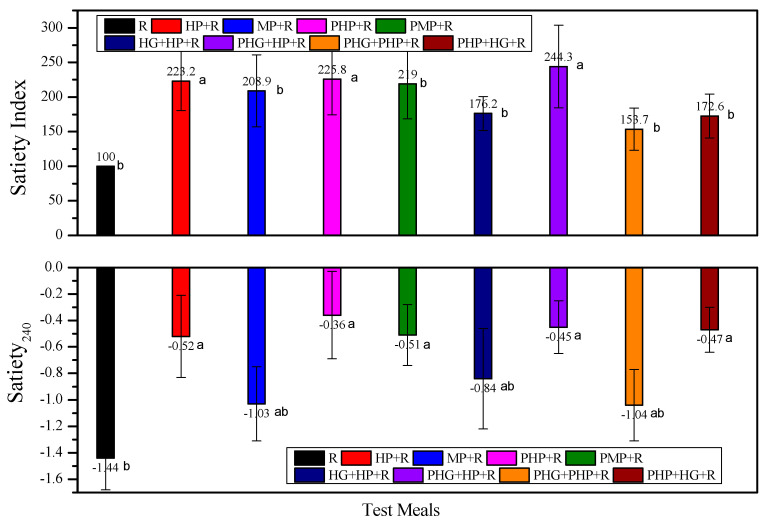
Satiety index and satiety at 240 min for test foods. R, rice; HP, highly cooked potato; PHP, preload highly cooked potato; MP, minimally cooked potato; PMP, preload minimally cooked potato; HP + R, co-ingestion of HP and rice; MP + R, co-ingestion of MP and rice; PHP + R, preload HP prior to rice meal; PMP + R, preload MP prior to rice meal; HG, partially hydrolyzed guar gum; PHG, preload HG; HG + HP + R, co-ingestion of HG, HP and rice; PHG + HP + R, preload HG prior to co-ingestion of HP and rice; PHG + PHP + R, co-preload of HG and HP prior to rice meal; PHP + HG + R, preload of HP prior to co-ingestion of HG and rice. Values are shown as the mean value with their standard errors represented by vertical bars. Data were analyzed by one-way (treatment) ANOVA with Duncan’s multiple range test, significant differences (*p* < 0.05) were represented by different letters and values are shown as the mean value with their standard errors represented by vertical bars.

As shown in Figure 5, in terms of SI and satiety increment at 240 min, all the mixed meals showed comparable or improved self-reported satiety compared with the rice reference. Compared with MP counterparts, most of the HP containing meals presented higher SI as well as higher satiety increment at 240 min. The incorporation of HG as preload showed higher SI and satiety increments than that of the HP + R mixed meals. There was no correlation between blood glucose iAUC_120–240_ and the satiety parameters.

## 4. Discussion

In this study, on the basis of equal available carbohydrates replacement, cooked potato as a preload to rice meal significantly cut the acute postprandial glycemic excursion in young healthy females, irrespective of the GI of the preload. The HP preload meal resulted in better GRs than its co-ingested counterparts, indicating that the timing and manner of high GI food intake did make a difference. Co-preload of partial hydrolyzed guar gum and highly cooked potato (PHG + PHP + R) resulted in improved acute GR in terms of peak glucose value and glycemic excursion compared with either HG preload or HP preload. The preload meals presented comparable or improved self-reported satiety.

As the study was designed to observe the impact of preload GI on acute postprandial GR and satiety, we attempted to eliminate as much confounders as possible, including the protein, fat, dietary fiber, and phytochemical [33,34,35,36] contents of the test meals. In light of the fact that potato foods could either be a good source of resistant starch or a food high in rapid digest starch, depending on the cooking treatment [13], we successfully manipulated the glycemic characteristics (either a high GI or a medium GI food) of potato samples by modifying the microwave treatment while keeping the nutrient composition unchanged. This GI manipulating strategy may be applied to future research on this aspect.

Inconsistent with one of our hypotheses, no significant difference in GRs occurred between HP + R and MP + R, or PHP + R and PMP + R. Though there was a sharp difference of GI value between the two potato samples (82.9 vs. 56.2). The possible discrepancy brought by MP and HP might be diminished by the large bulk of rice, as the potato only accounted for 30% of the total AC in the test meals. Nevertheless, an isocaloric introduction of MP to a rice meal could increase RS and dietary fiber intake and, thus, might benefit the metabolic disease prevention and management [37].

Compared with their co-ingestion counterparts, the PHP + R and PMP + R achieved a 1.0 mmol/L reduction of MAGE and significantly smaller iAUC_0–60_, but did not blunt the iAUCs beyond 60 min. There is the possibility that the high GI potato preload stimulated the early secretion of insulin and promoting the release of gut peptides such as glycagon-like peptide-1 (GLP-1) and gastric inhibitory peptide (GIP) [38]. A previous study reported the 20 g glucose solution preload did increase the pre-meal and post-meal insulin secretion [39]. However, another study found that a flavored rice congee preload of 25 g of available carbohydrate cut the peak glucose value by about 0.7 mmol/L compare with the water preload, while the GLP-1 level decreased significantly and insulin iAUC_0–150_ had only an insignificant increase [5].

In the study, the medium-GI potato preload performed equally effectively, if not more effectively than its high-GI counterpart, in terms of lowering the glycemic excursion. High RS carbohydrate food would release glucose slowly during digestion and improve the insulin sensitivity rather than eliciting more GLP-1 and insulin as high GI starch food did [40]. In an acute trial, adding resistant starch to standard meals failed to increase the postprandial level of either insulin or GLP-1 [41]. Therefore, the glycemic stabilizing effect of HP and MP could not be attribute to either GLP-1 or insulin release.

Previous studies reported that meals with low GI or high content of RS help to modulate the GRs and insulin responses in subsequent meal via second meal effect [42,43]. Animal research suggested that the RS may improve insulin sensitivity by mechanisms, such as weight reduction, positive changes in colon microorganism [44], and other possible pathway independent of microbiota [45]. However, the interval between potato preload and rice meal in the present study was only 30 min, which might be too short to significantly enhance the RS fermentation in colon, or to elicit a second-meal effect, as the peak of GIP concentration commonly appears within 30–60 min after food ingestion [46]. The underlined mechanism of the preload effect of both highly and moderately available carbohydrates is still to be investigated.

It is well established that soluble fibers such as guar gum and β-glucan could suppress the blood glucose surge after meal by delaying gastric emptying, reducing enzyme-substrate interaction, and hampering the absorption of glucose in intestine via a high viscosity mechanism [20]. However, the effect of preload dietary fibers and resistant starch on postprandial blood glucose was inconsistent [47,48].

Compared with the high molecular soluble fiber, such as guar gum and β-glucan, the HG had relatively low viscosity. According to a previous report, three months intake of 6 g HG at the beginning of each meal significantly decreased the postprandial GR and fasting insulin in both healthy and prediabetic subjects. However, the postprandial increment of plasma GIP and GLP-1 dropped instead of increased after HG intervention [24]. The researchers attributed these results to a delayed gastric emptying [49], reduced rate of glucose diffusion through the intestine [50], and the modulation of gut peptide hormones as well as an improved microbiota. However, the microbiota explanation could not be applied to the present study, as it was an acute feeding trial instead of a three-month intervention.

In this study, as a dietary fiber component added to a potato-and-rice meal, the glycemic effect of HG seemed to depend on the manner it was incorporated into the diet. Ten grams of HG had no significant glycemic impact to the co-ingested potato-and-rice meal, either as a preload, or as a co-ingested item. Only when both the HG and HP were given as a double preload, could the iAUC_0–120_ be lowered by 23%, while the maximum glycemic excursion dropped for more than 30% compare with that of the HP + R and R.

There is a possibility that the synergy of low-dose or low-viscosity soluble fiber and available carbohydrate made a difference in terms of glycemic impact. It is noticed that HG in combination with 5.0 g fructose [51], or 5.3 g β-glucan and 17.7 g of available carbohydrate, mitigated GR in acute tests [47]. As a caloric load rather than dietary fiber component could elicit a significant impact on gut hormone release [52], while the incretin hormones could only effectively induce insulin secretion in case of an elevated blood glucose level [53], an appropriate combination and best timing of sugar (or high GI food) and dietary fiber preload might facilitate the release of the hormones with a reduced blood glucose peak. This hypothesis can explain the result that the double preload of HG and HP achieved better glycemic effect than a single HG preload or a single HP preload in the present study.

Non-fried cooked potato was well known for its high satiety index [12]. It is not a surprise that, compared with the rice reference, most of the HP containing meals presented higher SI as well as higher satiety increment at 240 min. The satiety index had a significant correlation with the satiety increment at 240 min, which underlined the reliability of the satiety assessment.

High RS content in food is usually associated with enhanced satiety [54]. However, the MP did not perform any better than its high GI counterpart either in co-ingestion or preload treatments in terms of satiety assessed by VAS. The disparity between RS content of HP and MP was only 0.8 g, too small a difference to stimulate the release of gut peptides such as PYY and GLP-1 and make a difference of acute satiety in 240 min [55].

In a previous study, potatoes with high GI (GI = 93) and low GI (GI = 53) generated comparable satiety in normal weight healthy subjects [56]. One study reported that the RS had no effect on subjective appetite and subsequent food intake in the prediabetic subjects [57]. One possible explanation might be that the reduced available carbohydrate content canceled out the satiety effect of RS in the MP. Nevertheless, a high RS diet could reduce food intake without affecting the subject satiety/appetite rating [58,59]

It is interesting that while the PHG + PHP + R had the lowest glycemia among test meals, it did not result in the highest satiety score at 240 min. The incorporation of HG did not significantly enhanced the satiety increments of the HP + R mixed meals, though the HG was reported to be able to slow down the gastric emptying rate [60] and enhance satiety even at a low dose (<5 g/d) in healthy subjects [61]. The effect of unavailable carbohydrates and dietary fiber on satiety-related gut hormones was inconsistent in previous studies [62,63]. The divergent results could be partially due to the dose and source of the test RS or fiber, the length of intervention period, and the study design.

To our knowledge, this is the first study on the impact of potato or tuber preload on GR. Since potato food contributes substantially to dietary carbohydrate in most part of the globe, the investigation on GR of a potato-rice based meal is relevant. A latest published intervention study proved that the introduction of non-fried potato food resulted in better diet quality than its refined grain counterpart, without any detrimental glycemic effects [15]. However, the study did not investigate the preload effect of the potatoes. Secondly, it has not yet been investigated that if there is any difference between high GI and medium or low GI carbohydrate food in terms of the preload effect. We perfectly controlled the test meal composition and eliminated most confounding factors introduced by the discrepancy among food ingredients of the test meals by manipulating the GI and RS content with cooking treatment. Thirdly, the possible synergy effect of high GI preload and soluble fiber found in the study deserves further investigation.

The present study was conducted as an acute trial in healthy young female volunteers and the results needed to be confirmed by intervention trials in the obese, the prediabetic and the diabetic subjects. The number of subjects for the satiety study was limited. The insulin and related gastrointestinal hormones, such as GLP-1, GIP, and PYY, need to be investigated for a better understanding of the underlying mechanisms. In addition, whether the potato preload generated a lowered blood glucose peak via either a “mild-and-earlier” or a stronger pancreatic beta cell response is still to be confirmed.

## 5. Conclusions

In conclusion, our study found that within an equicarbohydrate exchange framework, taking non-fried cooked potato as preload to a high GI japonica rice meal could mitigate the postprandial glycemic excursion of a rice meal irrespective of the GI of the preload in young healthy females. The combination of HG and HP as double preload resulted in better GR than either single HG or HP preload did. Given the importance of glycemic homeostasis in chronic disease prevention [64] and the global high potato consumption, it seems that optimizing the manner of potato food ingestion might be a solution to some possible negative effects of potato food on metabolic consequence. 

## Figures and Tables

**Table 1:** Nutritional composition of administered test meals (per serving).

Test Meals	Rice (g)	Potatoes (g)	HG (g)	Weight (g)	Protein (g)	Fat (g)	Soluble Fiber (g)	Insoluble Fiber (g)	Available CHO (g)	Energy (kcal)
Glucose (25 g AC)	-	-	-	355.56	-	-	-	-	25.0	100.0
HP	-	110.0	-	355.56	3.4	0.1	0.9	1.6	25.0	114.5
MP	-	152.0	-	355.56	3.4	0.1	0.9	1.6	25.0	114.5
Glucose (50 g AC)	-	-	-	355.56	-	-	-	-	50.0	200.0
Rice	143.1	-	-	355.56	6.2	0.5	0.0	0.7	50.0	229.3
HP + R	100.2	66.0	-	355.56	6.4	0.4	0.5	1.4	50.0	229.2
MP + R	100.2	91.2	-	355.56	6.4	0.4	0.5	1.4	50.0	229.2
PHP + R	100.2	66.0	-	355.56	6.4	0.4	0.5	1.4	50.0	229.2
PMP + R	100.2	91.2	-	355.56	6.4	0.4	0.5	1.4	50.0	229.2
HG + HP + R	100.2	66.0	11.8	355.56	6.5	0.4	10.5	1.4	50.0	229.6
PHG + HP + R	100.2	66.0	11.8	355.56	6.5	0.4	10.5	1.4	50.0	229.6
PHP + HG + R	100.2	66.0	11.8	355.56	6.5	0.4	10.5	1.4	50.0	229.6
PHG + PHP + R	100.2	66.0	11.8	355.56	6.5	0.4	10.5	1.4	50.0	229.6

AC, available carbohydrate; CHO, carbohydrate; HP, highly cooked potato; PHP, preload HP. MP, minimally cooked potato; PMP, preload MP. R, rice; HP + R, co-ingestion HP with rice; MP + R, co-ingestion MP with rice; PHP + R, preload HP 30 min prior to rice ingestion; PMP + R, preload MP 30 min prior to rice ingestion; HG, partially hydrolyzed guar gum; PHG, preload HG. HG + HP + R, co-ingestion HG, HP and rice; PHG + HP + R, preload HG, with HP and rice co-ingested 30 min later; PHG + PHP + R, preload HP and HG, with rice ingested 30 min later; PHP + HG + R, preload HP, with HG and rice co-ingested 30 min later. Nutritional composition was determined according to national standards. The weight data of rice and potatoes are shown in a cooked condition and plain water was supplied for balancing the weight.

**Table 2:** Indices of glycemic variability for test meals in 240 min (mean values and standard errors (SE), n = 17).

	R		HP + R		MP + R		PHP + R		PMP + R	
	Mean	SE	Mean	SE	Mean	SE	Mean	SE	Mean	SE
∆Peak (mmol/L)	4.1 ^a^	0.2	4.3 ^a^	0.2	4.2 ^a^	0.2	3.7 ^a,b^	0.2	3.2 ^b^	0.2
∆Low (mmol/L)	−0.3^a^	0.1	−0.6 ^b^	0.1	0.3 ^a,b^	0.1	−0.3 ^a^	0.1	−0.2 ^a^	0.1
MAGE (mmol/L)	4.4 ^a,b^	0.2	4.9 ^a^	0.2	4.5 ^a,b^	0.2	3.9 ^b,c^	0.2	3.4 ^c^	0.2
−∆G/∆G (%)	67.5 ^a,b^	3.8	74.5 ^a,b^	5.2	79.6 ^a^	4.4	77.5 ^a^	5.4	61.1 ^b^	4.7
CONGA_1_	1.8 ^a^	0.2	2.0 ^a^	0.2	1.9 ^a^	0.2	2.2 ^a^	0.2	1.7 ^a^	0.2
iAUC_0–30_	50.4 ^a^	2.9	51.7 ^a^	3.5	52.5 ^a^	3.7	41.3 ^a,b^	2.9	37.2 ^b^	3.4
iAUC_0–60_	142.3 ^a^	11.3	150.9 ^a^	9.4	144.8 ^a^	9.9	127.6 ^a,b^	6.7	113.5 ^b^	9.0
iAUC_0–120_	230.4 ^a^	23.1	229.3 ^a^	18.4	213.5 ^a^	17.7	223.4 ^a^	19.0	214.0 ^a^	18.1
iAUC_120–240_	46.2 ^a^	10.0	15.6 ^b^	4.2	24.7 ^a,b^	8.3	36.6 ^a,b^	7.8	44.3 ^a^	9.7
iAUC_0–240_	276.7 ^a^	28.9	244.9 ^a^	20.2	238.2 ^a^	20.8	260.0 ^a^	21.8	258.3 ^a^	22.8

R, rice; HP, highly cooked potatoes; MP, minimally cooked potatoes. HP + R, co-ingestion of HP and rice; MP + R, co-ingestion of MP and rice; PHP + R, preload HP with rice; PMP + R, preload MP with rice. MAGE, the maximum amplitudes of glucose excursion; −∆G/∆G, baseline recovery 1 h after peak; CONGA1, continuous overall net glycemic action; iAUC, the positive increments under the curve of postprandial GRs. Values are the mean glycemic characteristics of test meals with their standard errors. Data were analyzed by one-way (treatment) ANOVA with Duncan’s multiple range test, significant differences among test meals were represented by different letters (*p* < 0.05).

**Table 3:** Indices of glycemic variability for test meals in 240 min (mean values and standard errors (SE), n = 17).

	R		HP + R		HG + HP + R		PHG + HP + R		PHP + R		PHP + HG + R		PHG + PHP + R	
	Mean	SE	Mean	SE	Mean	SE	Mean	SE	Mean	SE	Mean	SE	Mean	SE
∆Peak (mmol/L)	4.1 ^a^	0.2	4.3 ^a^	0.2	4.1 ^a^	0.3	3.7 ^a^	0.2	3.7 ^a^	0.2	3.6 ^a^	0.2	2.9 ^b^	0.2
∆Low (mmol/L)	−0.3 ^a,b^	0.1	−0.6 ^b^	0.1	−0.4 ^a,b^	0.1	−0.3 ^a,b^	0.1	−0.3 ^a,b^	0.1	−0.3 ^a,b^	0.1	−0.2 ^a^	0.1
MAGE (mmol/L)	4.4 ^a,b,c^	0.2	4.9 ^a^	0.2	4.5 ^a,b^	0.3	4.0 ^b,c^	0.3	3.9 ^c^	0.2	3.9 ^c^	0.2	3.1 ^d^	0.2
−∆G/∆G (%)	67.5 ^b^	3.8	74.5 ^a,b^	5.2	85.6 ^a^	4.2	68.8 ^a,b^	7.2	77.5 ^a,b^	5.4	71.7 ^a,b^	6.2	81.5 ^a,b^	5..4
CONGA_1_	1.8 ^b^	0.2	2.0 ^b^	0.2	1.9 ^b^	0.2	2.6 ^a^	0.2	2.2 ^a,b^	0.2	2.2 ^a,b^	0.2	1.7 ^b^	0.1
iAUC_0–30_	50.4 ^a^	2.9	51.7 ^a^	3.5	57.5 ^a^	3.8	5.5 ^c^	1.4	41.3 ^b^	2.9	40.3 ^b^	3.8	38.4 ^b^	3.2
iAUC_0–60_	142.3 ^a^	11.3	150.9 ^a^	9.4	151.7 ^a^	8.5	52.0 ^c^	4.6	127.6 ^a,b^	6.7	126.6 ^a,b^	7.6	106.0 ^b^	6.4
iAUC_0–120_	230.4 ^a^	23.1	229.3 ^a^	18.4	203.5 ^a^	13.5	197.3 ^a^	16.2	223.4 ^a^	19.0	228.9 ^a^	17.2	176.8 ^a^	15.2
iAUC_120–240_	46.2 ^a,b^	10.0	15.6 ^c^	4.2	23.8 ^b,c^	6.3	53.7 ^a^	8.9	36.6 ^a,b,c^	7.8	56.5 ^a^	11.0	36.6 ^a,b,c^	9.5
iAUC_0–240_	276.7 ^a,b^	28.9	244.9 ^a,b^	20.2	227.2 ^a,b^	16.0	251.0 ^a,b^	20.0	260.0 ^a,b^	21.8	285.3 ^a^	25.0	213.3^b^	21.7

R, rice; HP, highly cooked potato; PHP, preload HP; HG, partially hydrolyzed guar gum; PHG, preload HG; HP + R, co-ingestion of HP and rice; HG + HP + R, co-ingestion of HG, HP and rice; PHG + HP + R, preload HG 30 min prior to co-ingestion of HP and rice; PHP + R, preload HP 30 min prior to rice; PHG + PHP + R, co-preload HG and HP 30 min prior to rice; PHP + HG + R, preload HP 30 min prior to co-ingestion of HG and rice. MAGE, the maximum amplitudes of glucose excursion; −∆G/∆G, baseline recovery 1 h after peak; CONGA_1_, continuous overall net glycemic action; iAUC, the positive increments under the curve of postprandial GRs. Values are the mean glycemic characteristics of test meals with their standard errors. Data were analyzed by one-way (treatment) ANOVA with Duncan’s multiple range test, significant differences among test meals were represented by different letters (*p* < 0.05).
